# Effective and Apparent Diffusion Coefficients of Chloride Ions and Chloride Binding Kinetics Parameters in Mortars: Non-Stationary Diffusion–Reaction Model and the Inverse Problem

**DOI:** 10.3390/ma13235522

**Published:** 2020-12-03

**Authors:** Jerzy J. Jasielec, Jakub Stec, Krzysztof Szyszkiewicz-Warzecha, Artur Łagosz, Jan Deja, Andrzej Lewenstam, Robert Filipek

**Affiliations:** Faculty of Materials Science and Ceramics, AGH University of Science and Technology, Al. Mickiewicza 30, 30-059 Krakow, Poland; stec@agh.edu.pl (J.S.); szyszkin@agh.edu.pl (K.S.-W.); alagosz@agh.edu.pl (A.Ł.); deja@agh.edu.pl (J.D.); andrzej.lewenstam@gmail.com (A.L.); rof@agh.edu.pl (R.F.)

**Keywords:** diffusion–reaction model, chloride ions transport, cement-based materials, inverse problem, effective diffusion coefficient, chloride binding kinetics parameters

## Abstract

A non-equilibrium diffusion–reaction model is proposed to describe chloride transport and binding in cementitious materials. A numerical solution for this non-linear transport with reaction problem is obtained using the finite element method. The effective chloride diffusion coefficients and parameters of the chloride binding are determined using the inverse method based on a diffusion–reaction model and experimentally measured chloride concentrations. The investigations are performed for two significantly different cements: ordinary Portland and blast furnace cements. The results are compared with the classical diffusion model and appropriate apparent diffusion coefficients. The role of chloride binding, with respect to the different binding isotherms applied, in the overall transport of chlorides is discussed, along with the applicability of the two models. The proposed work allows the determination of important parameters that influence the longevity of concrete structures. The developed methodology can be extended to include more ions, electrostatic interactions, and activity coefficients for even more accurate estimation of the longevity.

## 1. Introduction

Concrete in reinforced structures has a dual role—it resists the compressive stresses caused by external loads and its own weight and protects the reinforcing steel from direct exposure to weather and environmental factors, which would otherwise almost instantly initiate corrosion. The level of protection provided by the reinforcing steel is related to the properties of the concrete itself, and is the result of the proportions of ingredients, the type of binder, and the type and amount of mineral additives used. Intensive impacts of chlorides from sources other than sea water on concrete are often encountered. This can include impacts on elements of civil engineering structures, such as viaducts, overpasses, or bridges. In order to ensure adequate protection of the reinforcing steel, depending on the intensity of the mentioned interaction, concrete with a certain cement content and a correspondingly low water/cement ratio has to be used [1].

In recent years, considering the necessity of using a binder characterized by low hydration heat, blast furnace cements, i.e., CEM III/A 32.5 (N, R) or CEM III/A 42.5 (N, R) (norm EN 197-1 [2]), have been used. In the road and bridge construction industry, sulfate resistance and a low alkali content are also required in order to prevent the alkali–silica reaction, which can result in the abnormal expansion and cracking of concrete [3,4]. However, real-world examples have shown that construction elements made of ordinary cements have sufficiently good properties. There is a need for a better understanding of the relationships between the properties of the mentioned cements and Portland cement (e.g., CEM I according to EN 197-1) across a wide range of steel reinforcement protection applications. Various contributions from hydration products and resulting differences in the microstructure of hardened cement paste influence the chemical, physicochemical, and physical properties of concretes [5,6].

### Selected Methods Used for Determination of Chloride Diffusion Coefficients

The salt ponding test (implemented as the standard AASHTO T259 [7]) and bulk diffusion test (implemented as the standard NT BUILD-443 [8] and later developed as ASTM C-1556-03 [9]) are examples of non-stationary pure diffusion tests in which the apparent diffusion coefficient (*D_app_*) is determined by fitting experimental curves of the chloride concentration to the suitable analytical solution of Fick’s second law [7,8,9,10,11,12,13]. Chloride transport in concrete- and cement-based materials has drawn much attention in the last two decades due to the fundamental problem of chloride-induced corrosion of rebars in concrete structures [14,15,16]. However, many commonly used models based on Fick’s second law still neglect the binding of chlorides when determining the chloride diffusion coefficient. A critical review of the existing experimental and analytical approaches can be found in [14,15,16,17].

The chemical binding of chlorides in concrete is a complex process that is affected by several factors, including the binder composition, w/c factor, degree of cement hydration, degree of reaction from the supplementary cementitious materials, concrete mix design properties (such as the water/binder (w/b) ratio), temperature, and alkalinity of the pore solution [18]. The salt type has a very significant influence on the deterioration of concrete [18,19]. CaCl_2_ reacts with portlandite to form calcium oxychloride, which leads to massive leaching and pH decay, resulting in steel corrosion [19,20,21]. Exposure to MgCl_2_ leads to the formation of a wide range of secondary products (brucite, Friedel’s salt, magnesium silicate hydrate M–S–H, magnesium oxychloride, and calcium oxychloride), which is accompanied by cracking and loss of mechanical properties due to massive changes in the concrete microstructure [19,22,23]. In this work, we focus on the exposure to NaCl, which has a less detrimental effect on concrete, because the pH value of the pore water remains relatively high, protecting steel against corrosion until the critical concentration of chlorides is reached.

The formation of Friedel’s salt and calcium oxychlorides provides a mechanism for chloride binding in concrete [24]. The traditional approach to modeling the chloride binding is through the use of experimentally determined chloride binding isotherms [18,25,26]. These isotherms are used either by assuming thermodynamic equilibrium between free and bound chlorides [25] or in the form of sink–source terms in the governing equation of the transport problem [27]. The chloride binding isotherms are described in detail in Section 3.1.

Other models use the thermodynamic calculations instead of reaction isotherms. Samson and Marchand [28,29] presented a numerical model in which the chemical reactions and ionic diffusion process are solved using the splitting method, whereby the transport step is followed by correction of the chemical reaction step by adjusting the solid phase composition to satisfy the equilibrium relations with proper equilibrium constants. The transport equations take into account the electrical coupling between the ions and the chemical activity effects. Their model was compared to experimental results, showing a good match with the measurements. The model was able to reproduce simultaneous chemical effects, such as precipitation of ettringite and gypsum, as well as dissolution of portlandite and decalcification of C–S–H gel. The authors compared two different techniques to account for binding mechanisms: dissolution–precipitation and ionic exchange processes. This comparison [29] focused on the formation of Friedel’s salt (3CaO·Al_2_O_3_·CaCl_2_·10H_2_O), which is the dominant phase formed in cementitious materials exposed to chloride solutions [24,30,31,32,33,34]. The dissolution–precipitation approach always results in fronts of chloride concentration, which is difficult to observe experimentally. On the other hand, the ion exchange model, based on monosulfate–Friedel’s salt interaction, gives a good correlation between the model and the measured chloride concentration profiles. It also predicts a simultaneous formation of Friedel’s salt, which was confirmed by X-ray diffraction (XRD) analyses on hydrated cement pastes [29].

The complex reaction processes that occur in cement materials include the ion exchange and subsequent formation of several phases of concrete, i.e., portlandite, C–S–H, monosulfate, ettringite, goethite, Friedel’s salt, and Kuzel’s salt. A proper description of these processes requires the usage of thermodynamic databases. Azad and co-workers [35,36] developed an interface allowing the modeling of multi-phase and multi-component transport processes coupled with complex chemical reactions (using a Gibbs energy minimization (GEMS) database). They also included the effect of temperature on the reactions of chlorides with unhydrated and hydrated cementitious materials. In their approach, the extended Nernst–Planck equation is solved using the finite element method, while in every time step of the time-marching algorithm, thermodynamic calculations are performed using GEMS in order to determine the thermodynamically feasible products, activity coefficients, chemical potentials, and other thermodynamic quantities, such as the pH, fugacity, and the redox state of the system. A similar approach has also been used by Tran et al. [37].

Most recently, Isgor and Weiss [38] presented a modeling framework that enables the use of measurements of electrical resistivity in conjunction with thermodynamic and transport modeling to predict the service life of concrete structures. The authors used the thermodynamic calculations to compute the pore solution chemistry and resistivity, pore volumes, formation factor, and reactions between the solid and ionic components of the cementitious matrix, such as chloride binding. The thermodynamically calculated binding isotherms are comparable to the experimentally determined counterparts only in the 100% ordinary Portland cement (OPC) case. However, the thermodynamic calculations overpredict the binding at high chloride concentrations for the slag and fly ash blended systems.

The models mentioned above can be used to predict the chloride transport properties based on the known concrete composition. The input data used for thermodynamic modeling contain the mill certificate data for the cementitious materials, mixture proportion data (e.g., water–binder ratio), kinetic information for the cement (degree of hydration), and data on supplementary cementitious material reactions for blended systems.

In this work, we present an alternative approach, namely a formulation and solution for the inverse problem related to determining the effective diffusion coefficients (*D_eff_*) of chlorides and the kinetic chloride binding parameters in cementitious materials based on the results of a non-stationary diffusion test. In our approach, the reaction–diffusion model, which takes into account non-equilibrium chloride binding, is used to formulate the inverse problem of chloride transport in saturated concrete samples (corresponding to experimental conditions) in order to establish an effective diffusion coefficient based on a non-stationary diffusion test.

The simplicity of this novel approach allows its application for the estimation of the longevity of concrete structures in cases where certain parameters of the cement (e.g., detailed information about its microstructure) are not known.

## 2. Materials and Methods

### 2.1. Characterization of the Samples and Storage and Sampling Conditions

The aim of the study was to assess the impact of the type of binder on the protective properties of reinforcing steel. Two cements significantly differing in composition and properties were selected:

1. Ordinary Portland cement—CEM I 42.5 R (norm EN 197-1 [2]);
2. Blast furnace slag cement—CEM III/A 42.5 N (norm EN 197-1 [2]).

The objective of the measurements made during the course of this study was to determine the diffusion coefficients of chloride ions for two mortars made from these cements.

Measurements were performed on mortar samples with weight proportions of sand (Kopalnia Surowców Mineralnych Dziergowice, Górażdże Heidelberg Cement Group, Dziergowice, Poland), cement (CEM I 42.5R—Górażdże Cement SA, Górażdże Heidelberg Cement Group, Chorula, Poland or CEM III 42.5N-HSR/NA—Górażdże Cement SA—EKOCEM, Górażdże Heidelberg Cement Group, Dąbrowa Górnicza, Poland), and water. Taking into account the composition requirements for mortar operating under the influence of chlorides, all samples were prepared with the same water-to-cement (w/c) ratio of 0.50, with the sand used to prepare all samples was from the same batch. Mortar cubes measuring 150 mm were cast in molds. The weight proportions for sand, cement, and water were 3:1:0.5. The weighed ingredients were mixed in a mixer (HL120, Hobart GmbH, Offenburg, Germany) for 180 s, and immediately after mixing the samples were formed by compacting the mortar on a vibrating table (B005, ToRoPol, Warsaw, Poland) for another 15 s. The sand used for the preparation of mortars was previously subjected to a drying process in a way that does not lead to its separation. The final compositions of the mortars used in this research are presented in Table 1.

The strengths of the prepared mortars were assessed after 2, 28, and 90 days of hardening and the absorbency properties were assessed after 28 and 90 days of curing in water. The porosity evaluation was performed using mercury intrusion porosimetry (MIP) using a PoreMaster 60 instrument from Quantachrome Instruments (Boynton Beach, FL, USA), with measurements covering the conventional pore diameter range of 3 × 10^−3^ to 200 µm. The flexural strength and compressive strength evaluation was conducted on samples with dimensions matching those outlined in EN 196-1 [39], which were formed in accordance with the procedure described in this standard. Absorbency was measured for 100 mm cubic samples. The test specimens for porosity tests were obtained from fragments of 40 × 40 × 160 mm beams, which were not damaged during direct measurement of the compressive strength after 90 days of curing. The results of these tests are shown in Table 2. Mortar cubic samples measuring 150 mm were prepared and protected against moisture loss during the first 28 days of storage.

### 2.2. Non-Stationary Diffusion Measurements

Individual mortar samples were analyzed to assess the chloride contents in sample cross-sections after two different exposure times. The aim was to obtain the concentration of chloride ions as a function of the distance from the surface of a cube, which makes it possible to assess the chloride ion diffusion coefficient after a certain exposure time in the samples using the established properties (e.g., type of cement, w/c ratio, type of sand, and curing conditions). Due to research protocol, especially regarding the time-consuming preparation of each cement-based sample, chloride content measurements were performed after different times of exposure.

Cuboid-shaped samples with two faces and with the casting material positioned at a considerable distance from the surface were exposed to chloride penetration perpendicular to the surface, which was immersed in a solution of NaCl. Sampling was done using a typical carbide-tipped drill with a diameter of 8 mm.

The chloride concentration profiles for the ordinary Portland cement CEM I 42.5 R samples were determined after 10 and 14 months of exposure to 3% NaCl water solution, while the chloride concentration profiles for blast furnace slag cement CEM III/A 42.5 N samples were determined after 8 and 11 months of exposure.

Samples were taken at different distances, with 6 mm space between points. Drilling was performed on both sides of each sample, thereby preparing two series of samples (A and B) that were subjected to chloride penetration tests separately and used to calculate the average value. Material acquired in the form of drill cuttings was for direct test samples. The samples were taken in the form of powder, therefore they did not require additional preparation for analysis.

The chloride content assessments were performed using the following procedure: First, 5 g of obtained cement powder was transferred to a tall 400 mL beaker, then 50 mL of distilled water and 50 mL of diluted (1:2) nitric acid solution were added. The obtained solution was heated to boiling point and kept at boiling temperature for 90 s. Afterwards, the mixture was removed from the heating device and 5 mL of silver nitrate was added. The solution was heated for another 90 s, then filtered through a coarse porous paper filter into a 500 mL conical flask. The beaker, glass rod, and paper filter were washed with highly diluted (1:100) nitric acid until the filtrate and the washing residue reached a volume of 200 mL. The obtained mixture was cooled down to below 25 °C. After the addition of 5 mL of the indicator solution (NH_4_Fe(SO_4_)_2_·12H_2_O + HNO_3_) and vigorous shaking, the mixture was titrated with the ammonium thiocyanate solution (concentration 3.8 kg/m^3^) until one drop of the titration solution produced a slight reddish-brown color, which remained after further shaking. The chloride content (as % of the sample mass) was calculated as:

(1) $$C_{tot}=\frac{1.773\times 5\times (V_{b}-V_{t})}{1000\times V_{b}\times m_{s}}\times 100=0.8865\times \frac{V_{b}-V_{t}}{V_{b}\times m_{s}},$$

where *m_s_* is the mass of the test sample (i.e., 5 g), *V_t_* is the volume (in ml) of the ammonium thiocyanate solution used for the titration of the test solution, and *V_b_* is the volume (in ml) of the ammonium thiocyanate solution used for the blank titration. The procedure described above follows the CEN-EN 196-2 procedure [40].

The test results obtained for different exposure times presented in Table 3 and Table 4 indicate the chloride diffusion progress, showing changes of chloride concentration as a function of the distance from the surface contact with the NaCl water solution.

## 3. Modeling of Chloride Transport

### 3.1. Binding of Chlorides in a Mortar

Chloride ions can be captured and immobilized by the hydration products of a cement [6,41]. This process, known as chloride binding, can take place either through a chemical reaction (with calcium aluminate hydrates) or physical adsorption (on the surface of the C–S–H gel) [42,43,44]. In practice, it is very difficult to distinguish between chlorides that are physically or chemically bound in mortar, thus the total amount of bound chlorides is usually investigated [45]. Only free chlorides are able to diffuse in the mortar, while bound chlorides are immobile [14,46].

Many extensive reviews on this topic can be found in the literature [14,26,47,48]. The amount of bound chlorides increases with the increase of the free chloride concentration. This relationship is described using chloride binding isotherms:

(2) $$C_{b}=f(c_{f})$$

where *C_b_* is the bound chloride concentration ${(\mathrm{kg}}_{{\mathrm{Cl}}^{-}}{/\mathrm{kg}}_{\mathrm{solid}})$ and $c_{f}$ is the free chloride concentration (kg/m^3^). The commonly used isotherms are the Langmuir, Freundlich, and linear isotherms [14].

The Langmuir isotherm assumes monolayer adsorption and has the following form [48]:

(3) $$C_{b}=\frac{K_{b}\cdot c_{f}}{1+\beta \cdot c_{f}}$$

Where *K_b_* and β are constants, which vary with the binder composition. This isotherm describes the adsorption of particles by the sorbent surface, which is characterized by energetically homogeneous sorption sites. Only monomolecular coverage of the adsorbent surface is assumed, while the desorption rate from a particular sorption site is thought to be independent of the occupancy of the neighbouring sorption sites [49].

The assumptions required for the derivation of the Langmuir adsorption isotherm in the case of an ion exchange reaction are [50]:

1. The adsorbed ions must cover all of the adsorption sites at all times and must form no more than a monolayer on these sites;
2. The adsorption sites must be uniform and localized. The adsorbed ions, regardless of species, must not interact with one another;
3. The ion exchange process must be the replacement of a single ion by another single ion.

The Langmuir isotherm has been used to describe the relationship between free and bound chlorides in cement-based materials in several papers [42,51]

Some authors [42,45,47,52,53] have shown that the experimental data for the chloride binding in mortar obey the Freundlich isotherm across a wide range of free-chloride concentrations:

(4) $$C_{b}=K_{b}\cdot c_{f}{}^{\eta }$$

where *K_b_* is the binding capacity of mortar $\left(m^{3\eta }/kg^{\eta }\right)$ and η≥0 (dimensionless) is the binding intensity parameter. The Freundlich isotherm assumes monolayer adsorption onto sorbent surfaces, which are characterized by heterogeneous sorption sites [49]. The Freundlich adsorption isotherm equation is rigorously derived for the trace adsorption of an ion participating in an exchange reaction. The assumptions required for a derivation of this isotherm in the case of a binary exchange reaction are [54]:

1. The adsorption sites may be grouped into classes, each of which is characterized by the number of sites it contains and by the relative affinity it possesses for the exchanging species.
2. For each class of sites, exchange is described by the competitive Langmuir equation (all assumptions for the Langmuir isotherm must be met).

Finally, the linear isotherm is applicable in several cases [43,55,56]:

(5) $$C_{b}=K_{b}\cdot c_{f}$$

The linear relationship overestimates chloride binding at high chloride concentrations and underestimates it at low chloride concentrations [48]. This isotherm (also referred to as the Henry isotherm) cannot be thermodynamically deduced, and therefore caution must be exercised when it is applied [49]. The above is an oversimplification that seems to be applicable only within a limited range of chloride concentrations [52]. Notice that the linear isotherm is a special combination of both the Langmuir isotherm (when *β* = 0) and Freundlich isotherm (when *η* = 1).

### 3.2. Diffusion–Reaction Model of Chloride Transport in Cementitious Materials

Let us consider a cementitious material sample. The free and bound chloride concentrations $c_{f}{\;(\mathrm{kg}}_{{\mathrm{Cl}}^{-}}{/m}^{3}{}_{\mathrm{solution}})$ and $C_{b}{\;(\mathrm{kg}}_{{\mathrm{Cl}}^{-}}{/\mathrm{kg}}_{\mathrm{solid}})$ are governed by mass conservation laws and Fick’s flux. No chloride is present at the beginning in the sample (initial condition) and a semi-infinite 1D geometry with Dirichlet boundary conditions is assumed. The model takes into account the diffusion of free chlorides, the chloride binding reaction (*r*), and the porosity of the material (*φ*). These factors produce the following set of equations:

(6) $$\left\{ \begin{array}{l} \varphi \frac{\partial c_{f}}{\partial t}\;=\;D_{eff}\frac{\partial^{2}c_{f}}{\partial x^{2}}\;-\;r\\ (1-\varphi )\rho_{s}\frac{\partial C_{b}}{\partial t}\;=\;r \end{array}\right.$$

where *D_eff_* is the effective diffusion coefficient of chloride ions. The solid-state density in mortar $\rho_{s}$ (kg/m^3^) is calculated using the following relation:

(7) $$\rho_{s}\;=\;\frac{\rho_{c}-\varphi \cdot \rho_{w}}{1-\varphi }\;$$

where the density of the pore solution is assumed to be $\rho_{w}=1000{\;\mathrm{kg}/m}^{3}.$

Chloride binding kinetics are described by a reaction term *r* (kg m^−3^ s^−1^), which depends on the binding isotherms:

(8) $$r=k\left[c_{f}-\frac{C_{b}}{K_{b}-\beta C_{b}}\right]\;\mathrm{for}\;\mathrm{the}\;\mathrm{Langmuir}\;\mathrm{isotherm},$$

(9) $$r=k\left[c_{f}-{\left(C_{b}/K_{b}\right)}^{1/\eta }\right]\;\mathrm{for}\;\mathrm{the}\;\mathrm{Freundlich}\;\mathrm{isotherm},$$

(10) $$r=k\left[c_{f}-C_{b}/K_{b}\right]\;\mathrm{for}\;\mathrm{the}\;\mathrm{linear}\;\mathrm{isotherm},$$

where constant *k* (s^−1^) is the binding reaction rate constant.

The concentrations of free chlorides at the boundaries follow the experimental condition of the test:

(11) $$c_{f}(0,t)=c_{0}=\mathrm{const},\;c_{f}(\ell ,t)=0\;$$

with $c_{0}=18.2{\;\mathrm{kg}/m}^{3}$ (3% NaCl) of the water solution in contact with the sample and ℓ is the width of the sample.

At the beginning, no chlorides are present in the sample:

(12) $$c_{f}(x,0)=0,\;C_{b}(x,0)=0\;$$

The total chloride concentration (%) is related to the free and bound chlorides concentrations by the following Equation:

(13) $$C_{tot}(x,t)\;=\;\frac{\varphi \;c_{f}(x,t)+(1-\varphi )\;\;\rho_{s}\;C_{b}(x,t)}{\rho_{c}}\;$$

The set of partial differential equations (PDE) (Equations (6)–(12)) was solved using the finite elements method (FEM) using COMSOL Multiphysics (version 5.4, COMSOL Inc., Burlington, MA, USA). In all calculations below, the density of the liquid-saturated mortar $\rho_{c}$ and the porosity φ were taken from Table 2 and the width of the sample was ℓ=150 mm.

### 3.3. The Inverse Problem for the Diffusion–Reaction Model

The solution for the diffusion–reaction model (Equations (6)–(12)) gives the free and bound chloride concentration profiles. Using Equation (13), the total chloride concentration can be calculated. Based on the experimentally measured total chloride concentrations, one can define the goal function, namely the difference between calculated and measured total chloride concentrations. In the diffusion–reaction model, the goal function *G_f_* is a function of three parameters for the linear isotherm and four parameters for Langmuir and Freundlich isotherms:

(14) $$\begin{array}{ll} Gf(D_{eff},k,K_{b},\beta )=\sum_{i=1}^{r}{\left(C_{i}^{\exp}-C_{tot}(D_{eff},k,K_{b},\beta )\right)}^{2} & \mathrm{for}\;\mathrm{Langmuir}\;\mathrm{isotherm},\\ Gf(D_{eff},k,K_{b},\eta )=\sum_{i=1}^{r}{\left(C_{i}^{\exp}-C_{tot}(D_{eff},k,K_{b},\eta )\right)}^{2}\;\; & \mathrm{for}\;\mathrm{Freundlich}\;\mathrm{isotherm},\\ Gf(D_{eff},k,K_{b})=\sum_{i=1}^{r}{\left(C_{i}^{\exp}-C_{tot}(D_{eff},k,K_{b})\right)}^{2}\;\; & \mathrm{for}\;\mathrm{linear}\;\mathrm{isotherm}. \end{array}$$

The optimization of these goal functions allows us to find the values of parameters *D_eff_*, *k*, *K_b_*, η and β, which give the best fit between the calculated total concentration profiles and experimental profiles.

## 4. Results

### 4.1. Optimization for a Single Measurement

Optimization was performed using the coordinate search method [57]. The parameters were restricted to intervals: $D_{eff}\in [10^{-13},\;10^{-11}],$ $k\in [10^{-8},\;10^{-6}],$ $K_{b}\in [10^{-5},\;10^{-2}],$ β∈[0, 1], and η∈[0, 1]. The results of the optimization for CEM I 42.5 R and CEM III/A 42.5N mortars are presented in Table 5, Table 6 and Table 7.

**Figure 1:**
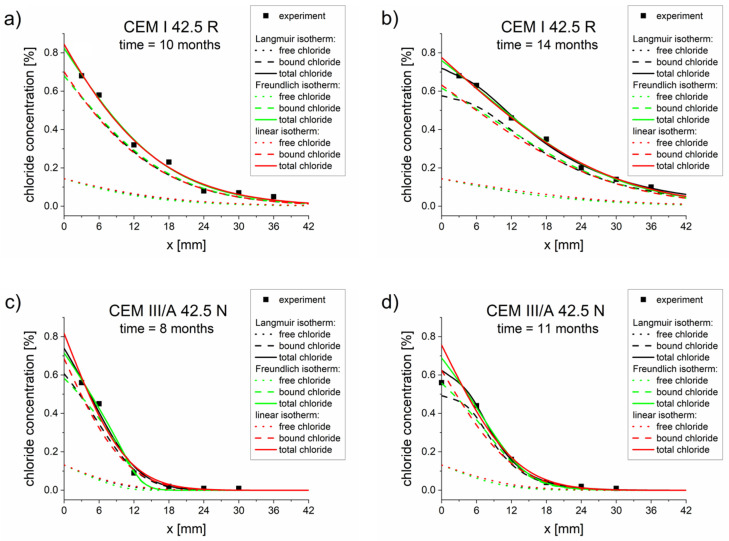
Chloride concentrations profiles obtained from experiments (points) and computations (lines) using a diffusion–reaction model with different binding isotherms and parameters *D_eff_*, *k, K_b_, η,* and *β*, optimized for a single measurement (Table 5, Table 6 and Table 7): (**a**) CEM I 42.5 R–10 months, (**b**) CEM I 42.5 R–14 months, (**c**) CEM III/A 42.5 N–8 months, (**d**) CEM III/A 42.5 N–11 months.

The comparison of the experimental concentration profiles and the profiles calculated for optimized parameters (Table 5, Table 6 and Table 7) are presented in Figure 1. Good agreements between the experimental total chloride concentration (points) and calculated concentration profiles (lines) were observed for both mortars and chloride exposition times.

### 4.2. Optimization Based on Two Measurements

In the diffusion–reaction model, the chloride diffusion coefficient is an effective diffusion coefficient, which unlike apparent diffusion coefficient (for simple diffusion model) does not depend on time [14,15,16]. Consequently, the goal functions can be defined for all measured times together:

(15) $$\begin{array}{l} Gf(D_{eff},k,K_{b},\beta )=\frac{1}{n}\sum_{j=1}^{n}\sum_{k=1}^{r}\left({\left(C_{k}^{\exp}(t_{j})-C_{tot}(D_{eff},k,K_{b},\beta ,t_{j})\right)}^{2}\right)\;\;\mathrm{for}\;\mathrm{Langmuir}\;\mathrm{isotherm},\\ Gf(D_{eff},k,K_{b},\eta )=\frac{1}{n}\sum_{j=1}^{n}\sum_{k=1}^{r}\left({\left(C_{k}^{\exp}(t_{j})-C_{tot}(D_{eff},k,K_{b},\eta ,t_{j})\right)}^{2}\right)\;\;\mathrm{for}\;\mathrm{Freundlich}\;\mathrm{isotherm},\\ Gf(D_{eff},k,K_{b})=\frac{1}{n}\sum_{j=1}^{n}\sum_{k=1}^{r}\left({\left(C_{k}^{\exp}(t_{j})-C_{tot}(D_{eff},k,K_{b},t_{j})\right)}^{2}\right)\;\;\;\;\;\mathrm{for}\;\mathrm{linear}\;\mathrm{isotherm}. \end{array}$$

We performed diffusion experiments for two different times (*n* = 2 in Equation (15)), and optimization of the goal function (Equation (15)) was also carried out using the coordinate search method, with parameters sought for the following intervals:$D_{eff}\in [10^{-13},\;10^{-11}],$ $k\in [10^{-8},\;10^{-6}],$ $K_{b}\in [10^{-5},\;10^{-2}],$ β∈[0, 1], η∈[0, 1].

**Figure 2:**
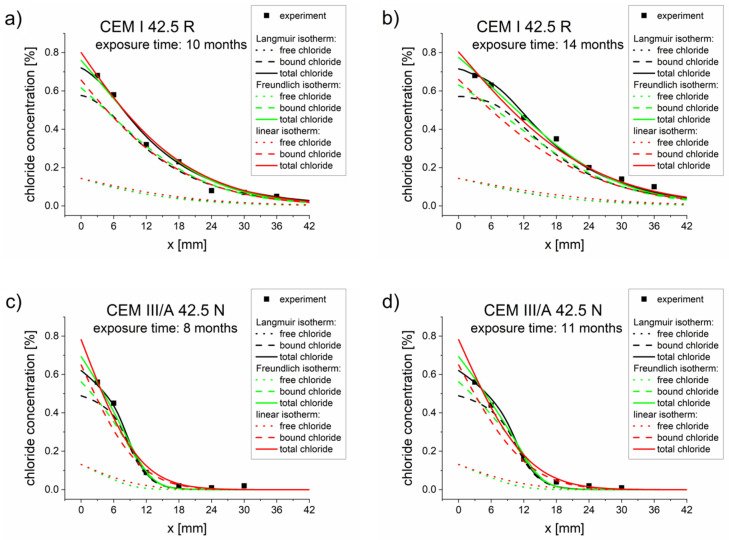
Chloride concentrations profiles obtained from experiments (points) and calculated using the diffusion–reaction model with different binding isotherms and parameters *D_eff_*, *k, K_b_, η,* and *β*, optimized for two measurement times (Table 5, Table 6 and Table 7). (**a**) CEM I 42.5 R–10 months, (**b**) CEM I 42.5 R–14 months, (**c**) CEM III/A 42.5 N–8 months, (**d**) CEM III/A 42.5 N–11 months.

The optimization results are presented in Table 5, Table 6 and Table 7. The comparisons between the experimental and simulated concentration profiles obtained for optimized parameters (Table 5, Table 6 and Table 7) are presented in Figure 2. Good agreements between the experimental values (points) and simulated concentration profiles were observed.

As can be appreciated from the values contained in Table 5, Table 6 and Table 7, diffusion coefficients computed on the basis of one and two measurements give similar results, with the two-measurement values usually being close to the average of the single-measurement values. However, at this point we cannot judge with certainty which coefficient obtained through the optimization is better (closer to reality), as this would require a detailed mathematical analysis of the inverse problem defined by Equations (15), (14), (6) and (9). We can only resort to the general view that statistical information extracted from a greater sample are usually better than extracted from a smaller sample.

Moreover, the microstructure of the cement matrix changed over time. This was especially the case for CEM I 42.5 R, where the *D_eff_*, *k*, *K_b_*, and *β* values differed for times *t*_1_ and *t*_2_. The most pronounced difference was noted for the Langmuir isotherm (Table 5).

### 4.3. Apparent and Effective Diffusion Coefficients

The effective diffusion coefficient *D*_eff_ obtained from the diffusion–reaction model describes the transport properties of the pore system of a water-saturated mortar. The apparent diffusion coefficient *D_app_,* calculated using a simplified diffusion model (Appendix A), characterizes (or tries to do so) the overall effect of chloride ion transport by accounting for two processes: free ion movement (in the pore system) and ion binding (at the surfaces of the pores). The effective and apparent diffusion coefficients are in some works related by the following formula [14]:

(16) $$D_{app}=\frac{D_{eff}}{1+\frac{\partial C_{b}}{\partial c_{f}}}$$

or in the form $D_{app}=D_{eff}/R_{d}$ with $R_{d}=1+\partial C_{b}/\partial c_{f}$, called the retardation factor, which is meant to account for sorption or binding of ions to solids [58] (also see Appendix B). Hence, the concentration-dependent apparent diffusion coefficient for the respective isotherms (shown in Equations (3)–(5)) is:

(17) $$D_{app}=\frac{D_{eff}}{1+K_{b}{\left(1+\beta \;c_{f}\right)}^{-2}}\;\mathrm{for}\;\mathrm{the}\;\mathrm{Langmuir}\;\mathrm{isotherm},$$

(18) $$D_{app}=\frac{D_{eff}}{1+K_{b}\;\eta \;\;c_{f}^{\eta -1}}\;\mathrm{for}\;\mathrm{the}\;\mathrm{Freundlich}\;\mathrm{isotherm},$$

(19) $$D_{app}=\frac{D_{eff}}{1+K_{b}}\;\mathrm{for}\;\mathrm{the}\;\mathrm{linear}\;\mathrm{isotherm}.$$

**Figure 3:**
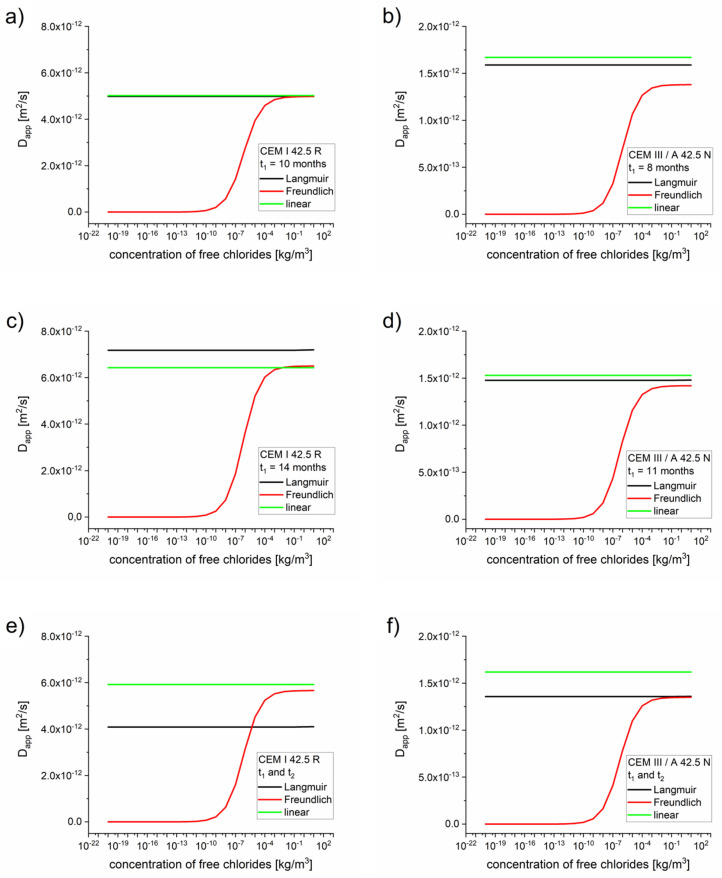
Apparent diffusion coefficients as functions of the concentrations calculated for optimized parameters *D_eff_*, *k, K_b_, η,* and *β*, optimized for a single measurement (Table 5, Table 6 and Table 7). (**a**) CEM I 42.5 R–10 months, (**b**) CEM III/A 42.5 N 8 months, (**c**) CEM I 42.5 R–14 months, (**d**) CEM III/A 42.5 N–11 months, (**e**) CEM I 42.5 R–two measurements, (**f**) CEM III/A 42.5 N–two measurements.

The apparent diffusion coefficients as functions of the concentrations obtained using the diffusion–reaction model are presented in Figure 3.

**Figure 4:**
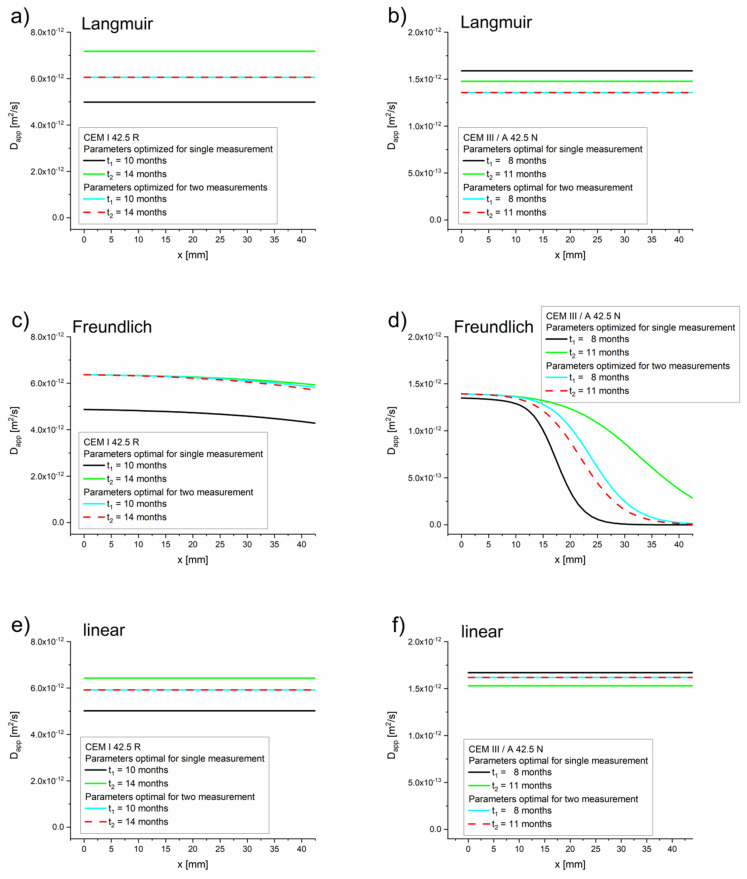
Apparent diffusion coefficients as functions of space obtained using Equation (16) and the optimal values of *D_eff_*, *k, K_b_, η,* and *β*, optimized for a single measurement (Table 5, Table 6 and Table 7) for Langmuir, Freundlich, and linear isotherms. (**a**) Langmuir isotherm–CEM I 42.5 R, (**b**) Langmuir isotherm–CEM III/A 42.5 N, (**c**) Freundlich isotherm–CEM I 42.5 R, (**d**) Freundlich isotherm–CEM III/A 42.5 N, (**e**) linear isotherm–CEM I 42.5 R, (**f**) linear isotherm–CEM III/A 42.5 N.

As expected, for high concentrations the apparent diffusion coefficient values equaled the effective diffusion coefficient values, $D_{app}=D_{eff}$ (see Table 5, Table 6 and Table 7). For low concentrations of free chlorides, the apparent diffusion coefficient values were significantly lower than the effective diffusion coefficient values. The dependence on η for the Freundlich isotherm is very strong due to expression $c^{\eta -1}$ (see Equation (18)). Therefore, the retardation of the chloride takes place mostly at the diffusion front, where the concentration of chloride is low (10^−3^ kg/m^3^ or even lower depending on *K_b_* and η), which is confirmed by the $D_{app}(x)$ function presented in Figure 4.

**Figure 5:**
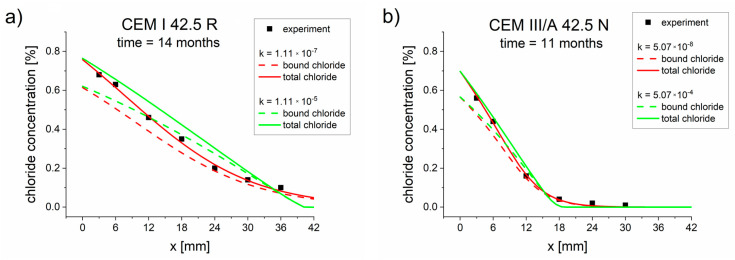
Calculated equilibrium (for *k*→ ∞) and non-equilibrium (*k* from Table 6) total and bound chloride concentrations for cements: (**a**) CEM I 42.5 R after 14 months and (**b**) CEM III/A 42.5 N after 11 months.

The above reasoning was carried out under the assumption of equilibrium values for binding chlorides, i.e., for the binding reaction rate constant k→∞ (see Appendix B). However, the concentrations calculated using the diffusion–reaction model (Figure 1 and Figure 2) were not equilibrium concentrations. To illustrate this point, Figure 5 compares the calculated equilibrium (for k→∞) and non-equilibrium (for *k* from Table 6) bound chloride concentrations, which clearly show that the bound chlorides are non-equilibrium, and consequently there is a need to use the diffusion–reaction model instead of the simplified relations (Equations (17)–(19)).

## 5. Discussion

### 5.1. Application of Binding Isotherms

Both ion exchange and adsorption may occur in solid−liquid systems. A strict differentiation between these two types of processes is impossible because of the presence of exchange and adsorption resins, which are used in liquid−solid adsorption applications [49]. However, the most common equilibrium models, such as the Langmuir and Freundlich isotherms, were derived by studying gas adsorption systems and then subsequently applied to solid−liquid systems [49].

It is a common misconception that only the adsorption of neutral molecules follows Langmuir or Freundlich equations. In order to apply these isotherms to ion exchange reactions such as chloride binding, the complete coverage assumption (adsorbed ions must cover all of the adsorption sites) is necessary. This condition did not apply in the original derivation of the Langmuir equation [59], which was developed to describe the adsorption of a gas by a clean solid. The failure to incorporate this important difference led Boyd et al. [60] to the erroneous conclusion that the Langmuir equation did not apply to ion exchange reactions. Details of the derivation of the Langmuir and Freundlich isotherms for ion exchange can be found in the work of Sposito [50,54]. The chloride binding in concrete has been successfully described in numerous papers using Langmuir [42,51] and Freunlich [42,45,47,52,53] isotherms.

There are several other isotherms that can be used to describe adsorption and ion exchange [49], including the Brunauer–Emmett–Teller (BET) isotherm, Tempkin isotherm, Redlich–Petersen isotherm, Tóth isotherm, and Fritz–Schülner isotherm. The possible application of these isotherms for the description of chloride binding in cement-based materials will be examined in our further work.

### 5.2. Influence of Porosity

In cementitious materials, pores can be simply classified into two groups, namely capillary pores (pore size/diameter *d* > 10 nm) and gel pores (*d* < 10 nm). According to the classification of pore sizes in hydrated cement pastes [61], capillary pores can be further divided into large capillaries (macropores, with *d* = 0.05 to 10 μm) and medium capillaries (large mesopores, with *d* = 10 to 50 nm), while gel pores can be further divided into small isolated capillaries (small mesopores, with *d* = 2.5 to 10 nm), micropores (with *d* = 0.5 to 3.5 nm), and interlayer spaces (with *d* ≤ 0.5 nm).

The w/c is probably the primary factor influencing the porosity of cement-based materials. It defines the spaces between cement grains during mixing and determines the initial capillary porosity of a sample. Products form throughout the hydration process, which progressively fill capillary pores, while the volume of the interlayer spaces increases [62].

In this work, we use mercury intrusion porosimetry (MIP), which is the well-established and thoroughly used technique for porosity measurements [63,64,65,66,67], despite its well-known limitations when applied to materials with irregular pore geometry, such as concrete [63,68,69]. MIP is widely used to study pore diameters ranging from 360 μm to less than 100 nm [63,68,70], but cannot provide information regarding gel pores (which remain non-intruded and are not quantified) or closed pores [68]. Other methods, such as backscatter scanning electron microscopy (SEM) analysis [71,72,73] and nuclear magnetic resonance (NMR) [62,74,75], can give more reliable results for the pore size distribution. A comparison between NMR relaxometry and MIP results [62] clearly showed that up to 75% of existing porosity is not visible when the MIP technique is used.

We performed the sensitivity analysis in order to check the influence of the porosity (as one of the models parameters) on the diffusion coefficients obtained using the inverse problem for the diffusion–reaction model. In our calculations, the porosity values increased 2-fold, 3-fold, and 4-fold compared to the results obtained using MIP (given in Table 2). We used the optimization method based on two measurements. The results are presented in Table 8, Table 9 and Table 10.

The sensitivity analysis clearly showed that the increase in porosity did not significantly influence the obtained effective diffusion coefficients (changes lower than 8% of the original value in all cases). This justifies the use of the MIP results in the presented analysis.

### 5.3. Further Extensions of the Model

The aqueous phase occupying a portion of the porous space is an ionic solution containing several ionic species (mainly OH^–^, Na^+^, K^+^, SO_2_^4−^, and Ca^2+^) [28]. The diffusion–reaction model presented in this work considers only the transport of chlorides. The influence of other ions on concrete transport is an obvious extension. This mutual influence reveals itself via electrostatic and chemical interactions. The latter would require the use of activity coefficients. Moreover, a study is currently being performed by our group, taking into account 3D microstructures of cement materials retrieved from the X-ray computed tomography. Finally, it should be stressed that the Fick flux or Nernst–Planck flux are only valid for dilute solutions, which is not the case for the cement pore solution. A concentrated electrolyte theory should be used for the flux, for example the Maxwell–Stefan equation.

## 6. Summary and Conclusions

The total chloride concentrations for two types of mortar samples at two exposure times in 3% NaCl aqueous solution were measured (experiment) and calculated (theory). The measured profiles were compared with the theoretical computations. A model that accounts for both diffusion and chloride ion binding reactions and which splits all chloride ions into the free and bound species was Equationted. This model was also the basis used to define the suitable inverse problem, which was solved using the coordinate search optimization method. As a result, the effective diffusion coefficient and kinetic parameters of chloride ion binding were obtained.

Three types of binding isotherms were analyzed, namely Langmuir, Freundlich, and linear isotherms. In most cases (except for the CEM III/A 42.5 N sample after 8 months), the best results were obtained for the Langmuir isotherm, as demonstrated by the smallest goal function value being obtained with this isotherm (Table 5, Table 6 and Table 7). The results obtained for Freundlich and linear isotherms were not as good as for the Langmuir isotherm but were still close to experimental results (Figure 1 and Figure 2). However, the differences produced by these three isotherms were not sufficiently pronounced to warrant a preference for one isotherm over the others. This may have been caused by the fact that the boundary chloride concentration was relatively low. In order to obtain adequate isotherm parameters and effective diffusion coefficients, it is very important to use high-quality total concentration profiles. The greater the number of points used for concentration probing, the better the fitting of the model and the more reliable the diffusion and binding parameters.

The diffusion–reaction model is closer to real chloride transport phenomena in mortar than the pure diffusion model (presented in Appendix A). Aside from the effective diffusion coefficient calculation, it also provides information on the chloride binding kinetics (*k*) and binding capacity (*K_b_*, *η*).

The effective diffusion coefficients determined for the CEM I 42.5 R samples were higher than the ones obtained for the CEM III/A 42.5 N samples. The results obtained for samples with a w/c ratio = 0.5 were in agreement with the results obtained by other authors [44], i.e., *D*_CEM I 42.5R_ = 2.9 × 10^−12^ m^2^/s (w/c = 0.45) to 5.7 × 10^−12^ m^2^/s (w/c = 0.6) and *D*_CEM III/A 42.5 N_ = 1.0 × 10^−12^ m^2^/s (w/c = 0.45) to 1.1 × 10^−12^ m^2^/s (w/c = 0.6).

**Figure 6:**
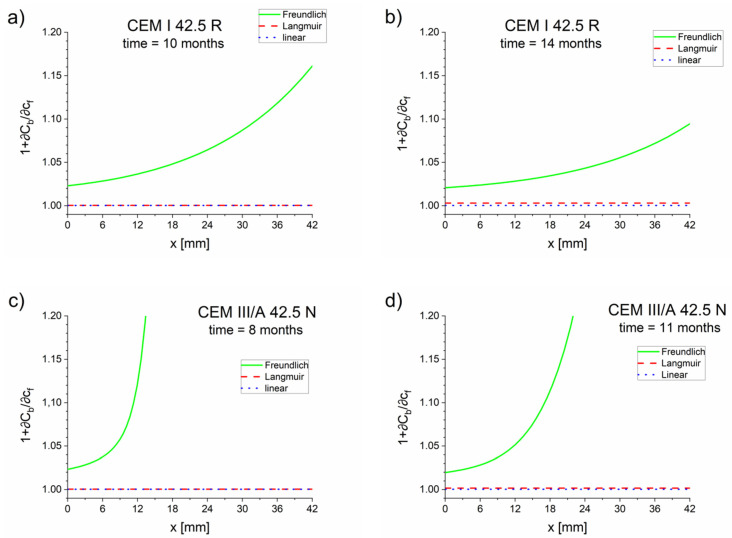
Retardation factor $\left(1+\partial C_{b}/\partial c_{f}\right)$ as a function of the positions of cements: (**a**) CEM I 42.5 R–10 months, (**b**) CEM I 42.5 R–14 months, (**c**) CEM III/A 42.5 N–8 months, (**d**) CEM III/A 42.5 N–11 months.

The apparent diffusion coefficient values estimated using the diffusion–reaction model (Equations (17)–(19)) indicated that the retardation of the diffusion process caused by the chloride binding occurred prominently with the Freundlich isotherm and mainly in the diffusion front (Figure 3 and Figure 6). For Langmuir and linear isotherms, the retardation coefficient was close to 1, meaning the apparent and effective chloride diffusion coefficients were practically equivalent.

A similar model accounting for the Freundlich binding isotherm and the non-equilibrium conditions between the free and bound chloride concentrations was proposed by Spiesz et al. [53] in order to describe chloride transport during a rapid chloride migration test. The value of the kinetic parameter *k* is much higher for a migration test than for a diffusion one (this work). The influence of the electric field is still not very well understood [48]. The presented model of chloride transport with reactions for both diffusion (this work) and migration tests (Spiesz et al. [53]) is an essential extension of a pure diffusion model based on Fick’s second law. However, both neglect the diffusion of other ions and their mutual interactions. These topics will be the subject of a separate paper.

## Figures and Tables

**Table 1:** Compositions of mortars (kg/m^3^) used for preparation of samples used in diffusion tests.

Type of Cement	Sand	Cement	Water
CEM I 42.5 R	1470	490	245
CEM III/A 42.5 N	1410	470	235

**Table 2:** Properties of the mortars used for the measurements.

Type of Cement	Bending Strength // Compression Strength (MPa)			Absorption (%)		Test Results from Mercury Porosimetry		
	Maturation Time			Maturation Time		Porosity (%)	Cumulative Pore Volume (mm^3^/g)	Density (kg/m^3^)
	2 Days	28 Days	90 Days	28 Days	90 Days			
CEM I 42.5 R	6.2 // 38.9	7.5 // 48.4	7.6 // 54.1	8.0	9.0	16.8	79.1	2130
CEM III/A 42.5 N	3.5 // 14.5	7.2 // 55.4	8.1 // 59.0	6.4	6.4	15.5	72.3	2140

**Table 3:** The chloride content in the mortar made using CEM I 42.5 R as a function of the distance to the surface after storing the samples in a 3% NaCl solution.

Time (Months)	Series	Chloride Content Measured (% of Sample Mass) at Depths of:						
		3 mm	6 mm	12 mm	18 mm	24 mm	30 mm	36 mm
10	A series	0.69	0.59	0.29	0.21	0.06	0.05	0.05
	B series	0.67	0.57	0.35	0.24	0.10	0.08	0.04
	**Average**	**0.68**	**0.58**	**0.32**	**0.23**	**0.08**	**0.07**	**0.05**
14	A series	0.67	0.62	0.46	0.35	0.20	0.13	0.10
	B series	0.68	0.64	0.46	0.34	0.20	0.14	0.10
	**Average**	**0.68**	**0.63**	**0.46**	**0.35**	**0.20**	**0.14**	**0.10**

**Table 4:** The chloride content in the mortar made using CEM III/A 42.5 N as a function of the distance to the surface after storing the samples in a 3% NaCl solution.

Time (Months)	Series	Chloride Content Measured (% of Sample Mass) at Depths of:					
		3 mm	6 mm	12 mm	18 mm	24 mm	30 mm
8	A series	0.56	0.44	0.07	0.01	0.01	0.00
	B series	0.55	0.45	0.11	0.02	0.01	0.01
	**Average**	**0.56**	**0.45**	**0.09**	**0.02**	**0.01**	**0.01**
11	A series	0.54	0.42	0.16	0.03	0.01	0.01
	B series	0.57	0.45	0.15	0.05	0.02	0.00
	**Average**	**0.56**	**0.44**	**0.16**	**0.04**	**0.02**	**0.01**

**Table 5:** Optimized parameters *D_eff_*, *k, K_b_*, and *β* for CEM I 42.5 R and CEM III/A 42.5N samples with the Langmuir binding isotherm applied.

Type of Cement	Time of Measurement (Months)	*D_eff_* (m^2^/s)	*k* (s^−1^)	*K_b_* (m^3^/kg)	*Β* (m^3^/kg)	Goal Function
CEM I 42.5 R	*t*_1_ = 10	4.99 × 10^−12^	9.09 × 10^−8^	5.69 × 10^−4^	0.02	3.44 × 10^−7^
	*t*_2_ = 14	7.20 × 10^−12^	3.38 × 10^−8^	3.16 × 10^−3^	0.46	1.14 × 10^−7^
	*t*_1_ and *t*_2_	6.08 × 10^−12^	4.10 × 10^−8^	3.30 × 10^−3^	0.48	4.48 × 10^−7^
CEM III/A 42.5 N	*t*_1_ = 8	1.59 × 10^−12^	9.98 × 10^−7^	5.69 × 10^−4^	0.03	3.70 × 10^−7^
	*t*_2_ = 11	1.48 × 10^−12^	7.75 × 10^−8^	1.67 × 10^−3^	0.26	1.47 × 10^−8^
	*t*_1_ and *t*_2_	1.36 × 10^−12^	4.14 × 10^−7^	1.66 × 10^−3^	0.26	1.62 × 10^−7^

**Table 6:** Optimized parameters *D_eff_*, *k, K_b_*, and η for CEM I 42.5 R and CEM III/A 42.5N samples with the Freundlich binding isotherm applied.

Type of Cement	Time of Measurement (Months)	*D_eff_* (m^2^/s)	*k* (s^−1^)	*K_b_* (m^3η^/kg^η^)	η (–)	Goal Function
CEM I 42.5 R	*t*_1_ = 10	4.98 × 10^−12^	5.16 × 10^−8^	1.83 × 10^−3^	0.51	3.48 × 10^−7^
	*t*_2_ = 14	6.50 × 10^−12^	5.07 × 10^−8^	1.58 × 10^−3^	0.50	1.34 × 10^−7^
	*t*_1_ and *t*_2_	5.66 × 10^−12^	5.70 × 10^−8^	1.61 × 10^−3^	0.50	5.00 × 10^−7^
CEM III/A 42.5 N	*t*_1_ = 8	1.38 × 10^−12^	8.41 × 10^−7^	1.55 × 10^−3^	0.48	1.47 × 10^−7^
	*t*_2_ = 11	1.42 × 10^−12^	1.11 × 10^−7^	1.43 × 10^−3^	0.50	4.40 × 10^−8^
	*t*_1_ and *t*_2_	1.35 × 10^−12^	3.34 × 10^−7^	1.52 × 10^−3^	0.48	2.36 × 10^−7^

**Table 7:** Optimized parameters *D_eff_*, *k,* and *K_b_* for CEM I 42.5 R and CEM III/A 42.5N samples with the linear binding isotherm applied.

Type of Cement	Time of Measurement (Months)	*D_eff_* (m^2^/s)	*k* (s^−1^)	*K_b_* (m^3^/kg)	Goal Function
CEM I 42.5 R	*t*_1_ = 10	5.02 × 10^−12^	1.26 × 10^−7^	4.26 × 10^−4^	3.56 × 10^−7^
	*t*_2_ = 14	6.43 × 10^−12^	9.38 × 10^−7^	3.77 × 10^−4^	1.60 × 10^−7^
	*t*_1_ and *t*_2_	5.92 × 10^−12^	1.63 × 10^−7^	3.93 × 10^−4^	5.76 × 10^−7^
CEM III/A 42.5 N	*t*_1_ = 8	1.67 × 10^−12^	9.88 × 10^−7^	4.06 × 10^−4^	6.39 × 10^−7^
	*t*_2_ = 11	1.53 × 10^−12^	9.63 × 10^−7^	3.70 × 10^−4^	1.50 × 10^−7^
	*t*_1_ and *t*_2_	1.62 × 10^−12^	7.30 × 10^−7^	3.85 × 10^−4^	4.96 × 10^−7^

**Table 8:** Optimized parameters *D_eff_*, *k*, *K_b_*, and *β* for CEM I 42.5 R and CEM III/A 42.5N samples with Langmuir binding isotherm applied.

Type of Cement	Porosity (%)	*D_eff_* (m^2^/s)	*k* (s^-1^)	*K_b_* (m^3^/kg)	*β* (m^3^/kg)	Goal Function
CEM I 42.5 R	16.8	6.08 × 10^−12^	4.10 × 10^−8^	3.30 × 10^−3^	0.48	4.48 × 10^−7^
	33.6	6.01 × 10^−12^	3.28 × 10^−8^	2.76 × 10^−3^	0.47	4.42 × 10^−7^
	50.4	5.89 × 10^−12^	2.41 × 10^−8^	2.03 × 10^−3^	0.43	4.69 × 10^−7^
	67.2	5.68 × 10^−12^	1.51 × 10^−8^	1.30 × 10^−3^	0.42	5.24 × 10^−7^
CEM III/A 42.5 N	15.5	1.36 × 10^−12^	4.14 × 10^−7^	1.66 × 10^−3^	0.26	1.62 × 10^−7^
	31.0	1.40 × 10^−12^	2.43 × 10^−7^	1.46 × 10^−3^	0.26	1.40 × 10^−7^
	46.5	1.46 × 10^−12^	1.54 × 10^−7^	1.21 × 10^−3^	0.26	1.81 × 10^−7^
	62	1.47 × 10^−12^	9.68 × 10^−8^	1.19 × 10^−3^	0.36	2.23 × 10^−7^

**Table 9:** Optimized parameters *D_eff_*, *k*, *K_b_*, and η for CEM I 42.5 R and CEM III/A 42.5N samples with Freundlich binding isotherm applied.

Type of Cement	Porosity (%)	*D_eff_* (m^2^/s)	*k* (s^−1^)	*K_b_* (m^3*η*^/kg*^η^*)	η (–)	Goal Function
CEM I 42.5 R	16.8	5.66 × 10^−12^	5.70 × 10^−8^	1.61 × 10^−3^	0.50	5.00 × 10^−7^
	33.6	5.70 × 10^−12^	4.25 × 10^−8^	1.48 × 10^−3^	0.48	5.01 × 10^−7^
	50.4	5.69 × 10^−12^	3.03 × 10^−8^	1.17 × 10^−3^	0.48	5.21 × 10^−7^
	67.2	5.60 × 10^−12^	1.80 × 10^−8^	8.39 × 10^−3^	0.45	5.58 × 10^−7^
CEM III/A 42.5 N	15.5	1.35 × 10^−12^	3.34 × 10^−7^	1.52 × 10^−3^	0.48	2.36 × 10^−7^
	31.0	1.42 × 10^−12^	1.76 × 10^−7^	1.47 × 10^−3^	0.44	2.49 × 10^−7^
	46.5	1.38 × 10^−12^	9.54 × 10^−7^	9.80 × 10^−4^	0.52	3.53 × 10^−7^
	62	1.46 × 10^−12^	5.85 × 10^−8^	1.92 × 10^−3^	0.14	1.92 × 10^−7^

**Table 10:** Optimized parameters *D_eff_*, *k,* and *K_b_* for CEM I 42.5 R and CEM III/A 42.5N samples with linear binding isotherm applied.

Type of Cement	Porosity (%)	*D_eff_* (m^2^/s)	*k* (s^−1^)	*K_b_* (m^3^/kg)	Goal Function
CEM I 42.5 R	16.8	5.92 × 10^−12^	1.63 × 10^−7^	3.93 × 10^−4^	5.76 × 10^−7^
	33.6	5.76 × 10^−12^	1.37 × 10^−7^	3.34 × 10^−4^	5.86 × 10^−7^
	50.4	5.46 × 10^−12^	7.12 × 10^−7^	2.52 × 10^−4^	6.47 × 10^−7^
	67.2	5.65 × 10^−12^	3.51 × 10^−8^	1.82 × 10^−4^	6.09 × 10^−7^
CEM III/A 42.5 N	15.5	1.62 × 10^−12^	7.30 × 10^−7^	3.85 × 10^−4^	4.96 × 10^−7^
	31.0	1.60 × 10^−12^	9.99 × 10^−7^	3.32 × 10^−4^	4.74 × 10^−7^
	46.5	1.56 × 10^−12^	9.99 × 10^−7^	2.72 × 10^−4^	4.69 × 10^−7^
	62	1.58 × 10^−12^	9.98 × 10^−7^	1.95 × 10^−4^	4.72 × 10^−7^

## Appendix A. Simplified Chloride Diffusion Model

Let us consider a sample made of mortar, inside which the changes of chlorides concentration *c*(*x*,*t*) in space and time are described by Fick’s second law (mass balance equation with Fick’s flux). No chloride is present at the beginning in the sample (initial condition) and a semi-infinite 1D geometry with Dirichlet boundary conditions is assumed:

(A1) $$\left\{ \begin{array}{l} \frac{\partial c}{\partial t}=D_{app}\frac{\partial^{2}c}{\partial x^{2}},\;\;\;\;for\;\;x\in (0,\infty ),\;\;t>0,\;\\ c(x,0)=0,\;\;\;c(0,t)=c_{0},\;\;c(\infty ,t)=0, \end{array}\right.$$

where *D_app_* is the apparent diffusion coefficient [14,15,16].

In the simplified diffusion model, it is assumed that all chloride ions are mobile. However, in real samples only free chlorides diffuse and the bound chlorides are immobile. This issue is addressed in the diffusion–reaction model presented in Section 3. Despite being vastly simplified, the model (Equation (A1)) is widely used. Detailed discussion of this topic can be found in recent reviews [14,15,16].

The model (Equation (A1)) has an analytical solution [76]:

(A2) $$c(x,t)=c_{0}\left(1-\mathrm{erf}\frac{x}{2\sqrt{D_{app}t}}\right)\;$$

The boundary concentration $c_{0}=1.82\%\cdot (\rho_{w}/\rho_{c})$ is set, where 1.82% is the concentration of chloride ions in a 3% NaCl solution, $\rho_{w}=1000\;{\mathrm{kg}/m}^{3}$ is the density of water, and *ρ*_c_ is the density of the mortar sample (see Table 2). Although Equation (A2) is a solution to a semi-infinite problem, for sample width ℓ→∞, it is a good predictor of finite-width systems, as long as $2\sqrt{D_{app}t}\ll \ell$ [77].

## Appendix B. Comments Concerning the So-Called Apparent Diffusion Coefficient

In the literature pertaining to chloride transport in concrete, the apparent diffusion coefficient, *D_app_*, also referred to as a non-steady-state diffusion coefficient, can be encountered. This transport parameter is described and used in various ways, but generally its role is to capture the time dependence of the transport properties. Hence, for example [17] recalls its connection with the effective diffusivity as:

(A3) $$D_{app}=D_{eff}/R_{d},$$

where $R_{d}$ is the retardation factor. If no sorption or binding of chloride occurs, then $R_{d}=1$. It is also noted that the apparent diffusion coefficient may vary with time, while the effective coefficient is constant.

The standard rationalization and quantification of this parameter is usually presented in the following manner. The mass balance equation of each species *I*,

(A4) $$\frac{\partial c_{i}}{\partial t}\;\;\;+\;\;\;\mathrm{div}\;J_{i}\;\;=\;\;r_{i},$$

plus constitutive laws for the flux $J_{i}$ and reaction term *r_i_* are used. The diffusive part of the flux is $-D_{i,eff}\nabla c_{i}$. Here, the parameter $D_{i,eff}$ is a true material property describing the rate of transport due to the concentration gradient—irrespective of side reactions, for instance. The balance of the mass distribution and flow is also influenced by the reactions included in Equation (A4) through the term *r_i_*. In general, the exact knowledge of a reaction term for any chemical reactions (either homogeneous or heterogeneous) is not a trivial matter, and ultimately it is equivalent to the detailed knowledge of the reaction mechanism. The simplified description of this reaction in the context of chloride binding can be quantified by the Equation $r_{i}=-\partial C_{b}/\partial t,$ where *C_b_* is the concentration of the bound chloride, which turns Equation (A4) into:

(A5) $$\frac{\partial c_{i}}{\partial t}\;\;\;+\;\;\;\mathrm{div}\;J_{i}\;\;=\;\;r_{i},$$

With the assumption on the form of the binding isotherm, $C_{b}=f\left(c_{i}\right)$ can be rewritten as:

(A6) $$\frac{\partial c_{i}}{\partial t}\;\;+\;\;\frac{\partial C_{b}}{\partial t}\;=\;\;\;-\mathrm{div}\;J_{i}$$

The notion of the apparent diffusion coefficient comes from this equation by placing factor $1+\frac{\partial c_{i}}{\partial C_{b}}$ inside the div operator. For example, in the case of the Nernst–Planck flux, $J_{i}=-D_{i,eff}\nabla c_{i}+z_{i}u_{i}c_{i}E$, this operation would yield $\frac{\partial c_{i}}{\partial t}\;\;=\;\;-\mathrm{div}\left[\frac{D_{i,eff}}{1+\frac{\partial c_{i}}{\partial C_{b}}}\nabla c_{i}+\ldots \right]$, hence the definition in Equation (A3) can be used. Of course, the reader will instantly recognize the shortcomings inherent in this development. First, it assumes that the binding reaction is instantaneous, meaning that the binding isotherm, which is strictly valid for the equilibrium state, is directly plugged into the kinetic equation (Equation (A4)) in the form of Equation (A5). While this approximation may in some cases be justified (fast binding), it is not universal [78,79]. Second, the following transformation,

(A7) $$\frac{1}{1+\frac{\partial c_{i}}{\partial C_{b}}}\mathrm{div}\left[D_{i,eff}\nabla c_{i}+\ldots \right]\;\;\;\to \;\;\;\mathrm{div}\left[\frac{D_{i,eff}}{1+\frac{\partial c_{i}}{\partial C_{b}}}\nabla c_{i}+\ldots \right]$$

is obviously mathematically not correct unless $\frac{\partial c_{i}}{\partial C_{b}}=const$, which is true only for the linear binding. Nevertheless, in some cases this may be viewed as a good approximation (see [80], especially comments pertaining to Equation (39)).

Of course, some authors use the reverse transformation, namely:

(A8) $$\frac{1}{1+\frac{\partial c_{i}}{\partial C_{b}}}\mathrm{div}\left[D_{i,eff}\nabla c_{i}+\ldots \right]\to \frac{D_{i,eff}}{1+\frac{\partial c_{i}}{\partial C_{b}}}\mathrm{div}\left[\nabla c_{i}+\ldots \right]$$

which is valid, but in that form one cannot claim that the expression $D_{i,eff}/(1+\frac{\partial c_{i}}{\partial C_{b}})$ is a well-defined material property, as it does not appear directly in the flux expression. As was pointed out in a recent review [17]: “it is not the instantaneous diffusion coefficient at a given exposure time but reflects a kind of an average effect at that time”.

Our understanding is that such a crux as the apparent diffusion coefficient is not necessary nowadays. It was contrived in the times when more accurate models were not available or were impractical due to the computing constraints. These limitations are not present now. Hence, in our opinion, the best way to describe the transport process of chloride in concrete is by using the true reaction terms with properly selected binding or sorption isotherms and to simulate the full system. This approach was adopted by [53,78] and was used to successfully produce materials and process parameters for non-steady transport.
